# Analysis of Growth Inhibition and Metabolism of Hydroxycinnamic Acids by Brewing and Spoilage Strains of *Brettanomyces* Yeast

**DOI:** 10.3390/foods4040581

**Published:** 2015-10-15

**Authors:** Michael Lentz, Chad Harris

**Affiliations:** Department of Biological Sciences, University of North Florida, Jacksonville, FL 32224, USA; E-Mail: chadlharris@hotmail.com

**Keywords:** *Brettanomyces*, hydroxycinnamic acid, fermentation, phenolic acid

## Abstract

*Brettanomyces* yeasts are well-known as spoilage organisms in both the wine and beer industries, but also contribute important desirable characters to certain beer styles. These properties are mediated in large part by *Brettanomyces*’ metabolism of hydroxycinnamic acids (HCAs) present in beverage raw materials. Here we compare growth inhibition by, and metabolism of, HCAs among commercial brewing strains and spoilage strains of *B. bruxellensis* and *B. anomalus*. These properties vary widely among the different strains tested and between the HCAs analyzed. Brewing strains showed more efficient metabolism of ferulic acid over *p*-coumaric acid, a trait not shared among the spoilage strains.

## 1. Introduction

*Brettanomyces* yeast are ubiquitous around the world and are commonly encountered in commercial fermentation settings. They are well-known for their ability to metabolize organic compounds common in wine must and beer wort into low-threshold volatile products that greatly affect the organoleptic qualities of the finished product. There are presently five species recognized within the genus *Brettanomyces*: *B. bruxellensis*, *B. anomalus*, *B. nanus*, *B. naardenensis*, and *B. custersianus*. Teleomorph (spore-forming) forms have been reported for *B. bruxellensis* and *B. anomalus* and are identified as the *Dekkera* genus [1]; these forms are rare and *Brettanomyces* will be used in this manuscript.

*Brettanomyces* is probably best known as a contributor to wine spoilage. The yeasts are distributed worldwide, and may arrive at a winery on fruit skins, or reside on winery surfaces or barrels [2,3,4,5,6]. Once present in a winery, they are difficult to eradicate. *Brettanomyces* are slow growing, and do not compete well with *Saccharomyces* yeast during active fermentation. Since they are tolerant of low pH and high alcohol, and can metabolize sugars and other organic molecules left behind by *Saccharomyces*, they may gain a foothold in a wine near the end of fermentation or during aging [2,7]. If they are allowed to persist, the wine may develop “Brett taint”, a characteristic set of off-flavors and aromas often described as rubber, burnt plastic, medicinal, leathery, goaty, barnyard, *etc.* *Brettanomyces*’ full metabolic contribution to wine flavor and aroma is poorly understood, however the characteristics described above are primarily attributed to these yeasts’ ability to metabolize hydroxycinnamic acids (HCAs) [8,9].

HCAs are a family of small phenolic acids found in all plant cells. They contribute to plant cell wall structure by forming cross-links between hemicellulose and lignin in the secondary cell wall [10,11]. Free HCAs are present in addition to crosslinks. Common HCAs include ferulic acid, *p*-coumaric acid, caffeic acid, and sinapic acid. They are present in varying proportions in different plant and cell types [12,13]. During maceration and vinification of grapes or malting and mashing of barley, HCAs are released from cells, and are always present in must and wort [14,15]. In addition to their role in plant cell wall structure, HCAs may also function as antimicrobial compounds in the plant, although the mechanisms are poorly characterized [11,16,17,18]. A variety of bacteria and fungi have evolved pathways to metabolize HCAs to less toxic products. In most of these organisms, HCAs are converted to vinyl derivatives through a phenolic acid decarboxylase (PAD) enzyme [19,20]. The specific vinyl compound depends on the HCA substrate, but all have very low flavor and aroma thresholds, and play a major role in wine spoilage. Some organisms, including *Brettanomyces*, encode a vinyl reductase (VR) enzyme that converts the vinyl compounds to ethyl derivatives, further contributing to off-flavors and aromas [21].

While *Brettanomyces* is nearly universally considered a flaw in wine, there are several styles of beer where *Brettanomyces*’ contribution is desired and even required. These styles include Lambics, gueuze, Flanders Red and Brown ales, and a growing list of American “wild” ales, whose popularity is rapidly increasing [22]. Some of the desired descriptors overlap with those in wine, including barnyard and leather. Other characteristics attributed to *Brettanomyces* include spicy, clove, and smoky [1,23]. The compounds that contribute to these beer qualities are also attributed to metabolism of HCAs by PAD and VR enzymes. The factors that determine the final qualities of the beer or wine are complex, and will depend on the type and relative concentration of HCA precursors, the timing of *Brettanomyces* introduction and relative proportion of *Brettanomyces* to other yeasts, and *Brettanomyces* strain variation [1,23]. *B. bruxellensis* is the dominant species associated with wine spoilage, while both *B. bruxellensis* and *B. anomalus* contribute to beer varieties [24,25]. Here we have investigated *Brettanomyces* strain variation for growth in and metabolism of different HCAs in order to better understand the complex contribution of *Brettanomyces* to commercial fermented beverages. The properties tested varied widely among the different strains analyzed and between three HCAs. Brewing strains of both species showed more efficient metabolism of ferulic acid over *p*-coumaric acid, a trait unique to these strains.

## 2. Experimental Section

### 2.1. Strains

All strains used were either *Brettanomyces bruxellensis* or *B. anomalus*. Eight strains were isolated from environmental sources. Strains 1–4, 6, and 7 were previously described isolates from ripe fruit [26]. Strains 5 and 8 are new isolates from Florida seagrape (*Coccoloba uvifera*) and blueberry (*Vaccinium* sp.), respectively. The remaining strains are commercially available for professional brewing and were obtained by purchase. All analyses started with single colony isolates. Commercial strains are deemed suitable for brewing as advertised. The environmental isolates were used to brew pilot batches and judged by experienced tasters [26]. Strain 6 has been used routinely by a microbrewery for 100% *Brettanomyces* fermentations. Brewing suitability is defined here as a yeast strain used as the sole fermenter of beer wort without detectable undesirable phenolic off-flavors or aromas. Strain information is shown in Table 1.

**Table 1:** Strain designation, source and suitability for brewing.

Strain	Species	Source *	Brewing Suitability
1	*B. bruxellensis*	Environmental (Bc02)	No
2	*B. bruxellensis*	Environmental (Bc07)	No
3	*B. bruxellensis*	Environmental (Bc11)	No
4	*B. anomalus*	Environmental (Cs01)	Yes
5	*B. anomalus*	Environmental (Cu02)	Yes
6	*B. anomalus*	Environmental (Ej02)	Yes
7	*B. anomalus*	Environmental (Rs01)	Yes
8	*B. anomalus*	Environmental (Vc01)	No
9	*B. anomalus*	Commercial (WLP645)	Yes
10	*B. bruxellensis*	Commercial (WLP650)	Yes
11	*B. bruxellensis*	Commercial (WLP653)	Yes
12	*B. anomalus*	Commercial (WY5151)	Yes

* WLP, White Labs Inc. (San Diego, CA, USA); WY, Wyeast Laboratories, Inc. (Odell, OR, USA).

### 2.2. Minimum Inhibitory Concentration

MYPG culture medium (3 g malt extract, 3 g yeast extract, 2 g Peptone, 10 g glucose per liter) was prepared containing zero through 20 mM ferulic acid, *p*-coumaric acid, or caffeic acid, in 2 mM increments. Caffeic acid and *p*-coumaric acid were also analyzed at 25, 30, and 35 mM concentrations. 200 µL aliquots were added to the wells of a sterile 96-well microtiter plate. The top two rows of eight columns contained zero mM, with increasing concentrations down the remaining wells in the column. 2 µL of actively growing yeast culture was added to rows 2–12, with each strain cultured in duplicate. The top row remained uninoculated (negative control). Plates were placed in ziploc bags with damp paper towels to prevent drying, and incubated at 26 °C for 72 h. Minimum inhibitory concentration (MIC) was defined as the lowest concentration of HCA in which no visible growth was observed.

### 2.3. Growth Characteristics in HCAs

MYPG culture medium was prepared with or without (control) 4 mM ferulic acid, *p*-coumaric acid, or caffeic acid. 15 mL samples were inoculated with cells to a concentration of 10^5^ cells/mL. Each strain was cultured in duplicate for each HCA and control culture. Cultures were grown at 24 °C on a shaker platform at 60 rpm. At regular time intervals over 14 days, 200 µL samples were removed and growth was measured by determining optical density at 600 nm. Each culture was sampled in duplicate at each time point.

### 2.4. Spectral Scan Peak Absorbance

MYPG was supplemented with 2 mM ferulic, *p*-coumaric, or caffeic acid. 20 µL of each prepared culture medium was diluted into 180 µL H_2_O. A spectral scan from 200–400 nm was performed on a BioTek PowerWave XS plate reader to determine peak absorbance wavelength and the absorbance value at the peak wavelength. This data was recorded for use in calculating HCA metabolism as described below. Peak absorbance corresponded linearly with HCA concentration (data not shown).

### 2.5. Metabolism of HCAs

MYPG was supplemented with 2 mM ferulic, *p*-coumaric, or caffeic acid. 200 µL of each MYPG/HCA preparation was added to wells of a 96-well microtiter plate. Two microliters of active 2–3 day culture of each yeast strain was added to a well; each strain in triplicate for each HCA. Plates were kept in a humidified ziplock bag and incubated at 26 °C for 14 days. After 14 days growth, samples were transferred to microtubes and cells were removed by centrifugation. 20 µL of culture supernatant was diluted into 180 µL H_2_O and the absorbance value at the peak wavelength was determined as described above for each HCA (313 nm for ferulic acid and caffeic acid; 287 nm for *p*-coumaric acid). The value for each sample was divided by the OD of the original sample, and the average determined for each strain, as well as the standard deviation from the mean. Results are reported as the concentration of HCA that remained in the sample after the 14 day incubation.

## 3. Results

### 3.1. Minimum Inhibitory Concentration

The MIC for each strain was tested in ferulic acid, *p*-coumaric acid, and caffeic acid from 0–20 mM. All strains grew well in media supplemented with caffeic acid at 20 mM, exhibiting no inhibition by this compound. Strains 1, 2, and 7–11 also grew at 20 mM *p*-coumaric acid. These strains represent both *B. bruxellensis* and *B. anomalus*, and brewing and non-brewing strains of each species. All strains were retested in 25, 30, and 35 mM *p*-coumaric and caffeic acid. All strains that grew in 20 mM *p*-coumaric acid were inhibited by 25 mM, and all strains were inhibited by 25 mM caffeic acid except 7 and 11, which required 30 mM for inhibition. Strains 3, 4, 5, 6, and 12 were unable to grow in *p*-coumaric acid concentrations of 10 mM, 12 mM, 8 mM, 14 mM, and 8 mM, respectively. Strain 3 is *B. bruxellensis*, while the rest are *B. anomalus*. Ferulic acid provided the strongest inhibition, with one strain (#4) growing in 14 mM, but no others growing at concentrations above 12 mM. Inhibition of growth above 6 mM was observed for three of the strains (3, 5 and 12). Growth at 12 mM but not above this concentration was seen for strains 6, 7, 9, 10 and 11, all strains that have been used successfully in pilot or commercial brewing. The remaining strains were inhibited by intermediate concentrations of ferulic acid (Table 2).

**Table 2:** Minimum inhibitory concentrations (millimolar) of hydroxycinnamic acids (HCAs) for each strain.

HCA	Strain											
	1	2	3	4	5	6	7	8	9	10	11	12
ferulic acid	10	10	8	14	8	12	12	16	12	12	12	8
*p*-coumaric acid	25	25	10	12	8	14	25	25	25	25	25	8
caffeic acid	25	25	25	25	25	25	30	25	25	25	30	25

### 3.2. Growth Properties

**Figure 1:**
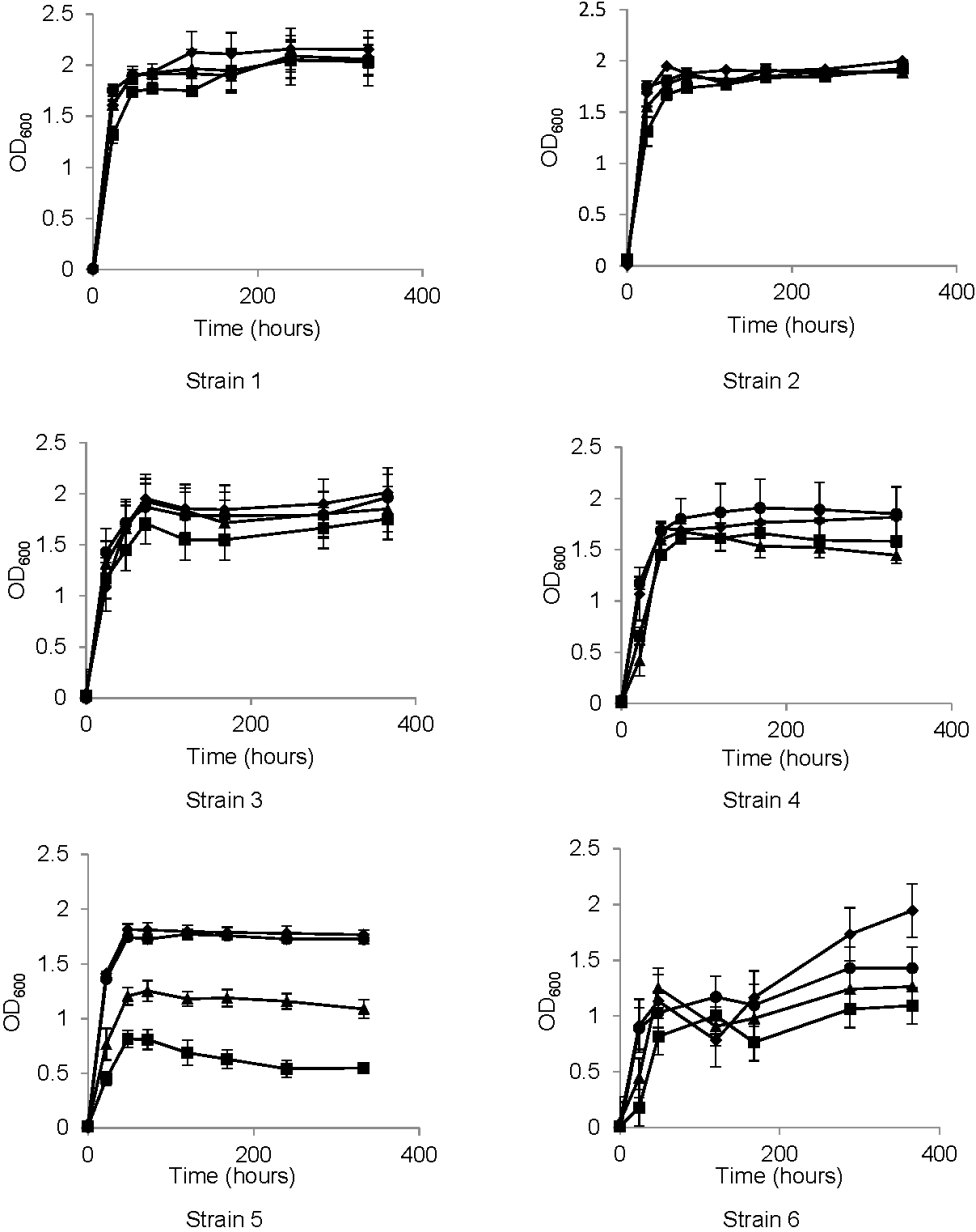

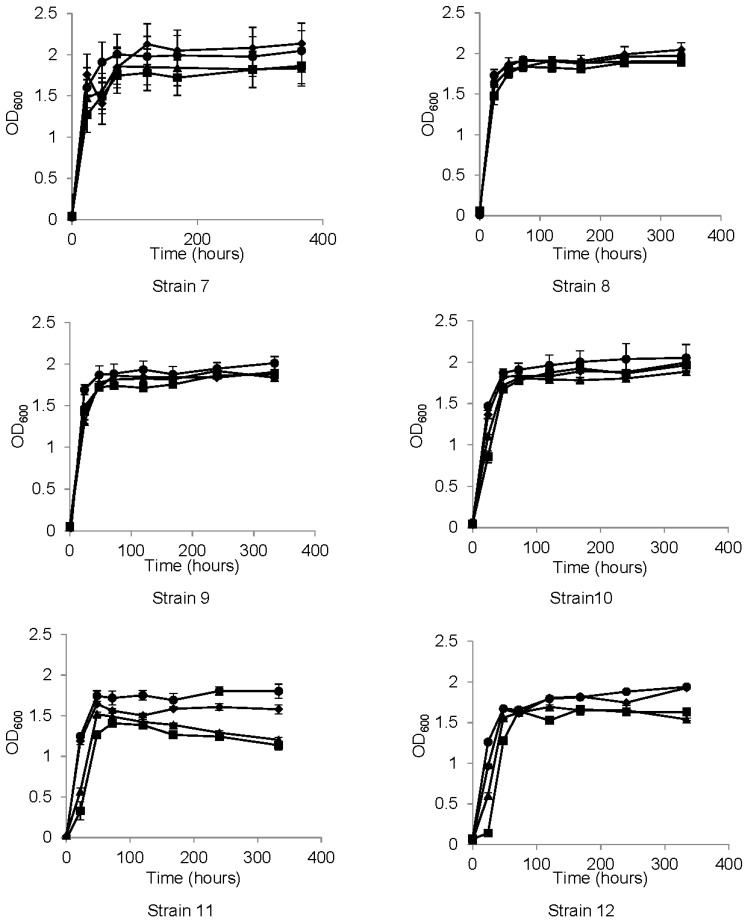
Growth curves for each strain in the presence or absence of added hydroxycinnamic acid (HCA). MYPG culture medium was supplemented with 4 mM HCA and growth measured by optical density at the indicated time points. Circles, control (no HCA); squares, ferulic acid; triangles, *p*-coumaric acid; diamonds, caffeic acid. Bars represent standard error of the mean.

We next determined what effect exposure to HCAs had on growth properties of *Brettanomyces* isolates. Cells were grown in the presence or absence of 4 mM HCA, and growth monitored by absorbance at 600 nm. There was no growth difference at any time point between control samples and cells grown in 4 mM caffeic acid, except for strain 11, which plateaued at a slightly lower density than control cells (Figure 1). In contrast, most strains were significantly impaired for growth in 4 mM ferulic acid and 4 mM *p*-coumaric acid. Some strains showed a growth lag at early time points, then caught up to the control cultures. Others showed a lag, but remained at lower cell densities throughout the growth period. Two isolates of *B. bruxellensis* and two of *B. anomalus* did not show any growth inhibition with any of the HCAs tested. Of those that were inhibited by ferulic and *p*-coumaric acid, all but two isolates were inhibited to the same extent by both acids, while two were inhibited to a significantly greater extent by ferulic acid than *p*-coumaric acid. Growth properties are summarized in Table 3.

### 3.3. Metabolism of HCAs

**Figure 2:**
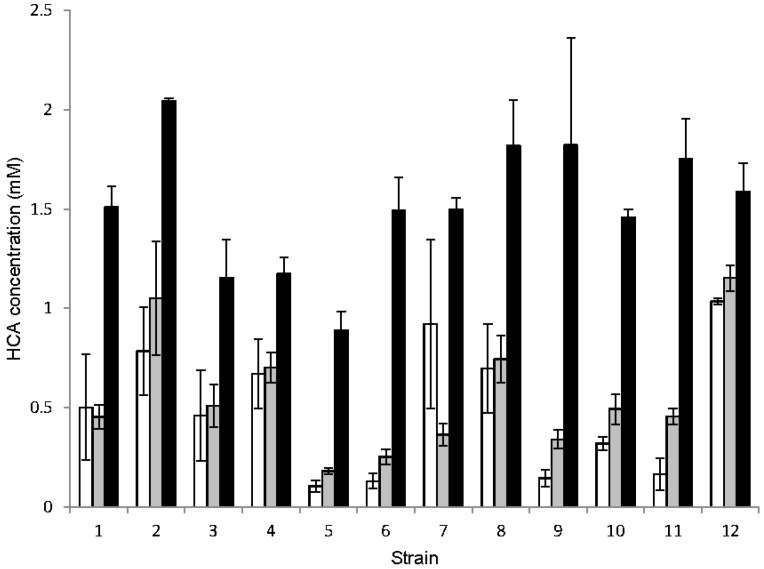
Metabolism of hydroxycinnamic acids (HCA) by yeast strains. Strains were incubated in MYPG supplemented with 2 mM HCA. The concentration of HCA remaining after 14 days was determined by measuring the absorbance at 313 nm (ferulic acid and caffeic acid) or 287 nm (*p*-coumaric acid) compared to the absorbance of a sample incubated without added cells. White bars, ferulic acid; gray bars, *p*-coumaric acid; black bars, caffeic acid. Graph represents mean plus standard error of the mean.

We next determined the relative metabolic activity of each strain for different HCAs. As can be seen in Figure 2, there is wide variation among isolates for metabolism of the different substrates. Some common themes were also observed. All 12 strains utilized caffeic acid least efficiently of the three HCAs tested, ranging from not using this substrate at all, to reducing the caffeic acid concentration by about 50 percent. Ferulic acid and *p*-coumaric acid were metabolized equally efficiently by five of the strains, however the HCA concentration remaining after metabolism ranged from 0.1 mM to approximately 1 mM (from a starting concentration of 2 mM) within this group. Six strains metabolized ferulic acid more efficiently than *p*-coumaric acid, again with wide strain-to-strain variation. Only one strain utilized *p*-coumaric acid more efficiently than ferulic acid. These results are further summarized in Table 3.

**Table 3:** Correlation of strain properties with brewing potential.

Strain	Greater inhibition by*			Metabolism of HCAs*			Suitability
	FA	*p*CA	Same	FA > *p*CA	*p*CA > FA	FA = *p*CA	For Brewing
1	X					X	No
2	X					X	No
3	X					X	No
4		X				X	Yes
5			X	X			Yes
6	X			X			Yes
7	X				X		Yes
8	X					X	No
9	X			X			Yes
10	X			X			Yes
11	X			X			Yes
12			X	X			Yes

## 4. Discussion

We have investigated variation among species and strain isolates of *Brettanomyces* yeast for metabolism of phenolic acids that naturally occur in beer wort and wine must. Isolates included commercial brewing strains and local environmental isolates. We aim to determine the role of strain variation in observed differences for production of volatile phenols in finished beer or wine. These compounds play an important role in wine spoilage, yet also contribute to unique, desired characteristics of certain beer styles.

Caffeic acid was the weakest inhibitor of the HCAs tested. This observation is in general agreement with published data for other strains that show weak or no inhibition of growth by this compound compared to other cinnamic acids [16]. Strains exhibited wide variability for inhibition by ferulic acid and *p*-coumaric acid. When a strain showed variation for inhibition by these two HCAs, ferulic acid was always a more potent inhibitor, with the sole exception of strain 4. This data supports results from similar experiments using different strains of *B. bruxellensis* and *B. anomalus* [27]. It appears that *Brettanomyces* in general are only weakly (if at all) inhibited by caffeic acid, and are slightly more susceptible to ferulic than *p*-coumaric acid. Additional strains should be tested to confirm this trend. MIC for the different HCAs could not be used to reliably distinguish between *B. anomalus* and *B. bruxellensis*, nor was there a notable distinction for this trait between strains successfully used in commercial brewing and those unsuitable for beer fermentation.

Growth properties in the presence or absence of HCAs varied among most of the strains tested, but without clear distinctions between species or between brewing *versus* spoilage strains. For ten of twelve strains tested, there was no growth inhibition in 4 mM caffeic acid compared to control cultures. This fits with the MIC data described above, where caffeic acid had the weakest inhibitory effect on all strains. Strains nine and twelve exhibited a slight but significant growth lag at 24 h when grown in caffeic acid, but no growth difference at remaining time points. Growth patterns for four strains (2, 3, 7 and 8) were identical to the control cultures for all three HCAs. This was expected for strain eight, which had the highest MIC for all three HCAs, but is unexpected for strain three which was completely inhibited by 8 mM ferulic acid and 10 mM *p*-coumaric acid in the MIC analysis. All of the other strains showed a significant growth lag in both ferulic acid and *p*-coumaric acid. In all but two strains where a lag was observed, treated cultures recovered and matched control treated growth levels within 72 h of inoculation. Strain five was the most severely inhibited in growth and also exhibited the lowest MIC for ferulic and *p*-coumaric acids. Interestingly, strain twelve showed identical MIC data as strain five, but was only minimally inhibited for growth in 4 mM HCAs. Strain 11 was inhibited similarly by both ferulic acid and *p*-coumaric acid, but not as severely as strain five.

Our growth data is broadly in line with work published by Harris, *et al.*, [28] using different *Dekkera* isolates. Our strains were less growth inhibited at 4 mM HCAs compared to the *Dekkera* isolates in 2 mM HCAs. Several of the *Dekkera* isolates also exhibited a growth lag with 2 mM caffeic acid, while our strains were consistently not inhibited by 4 mM of this HCA. Edlin *et al.* [21] also analyzed growth of one strain *B. anomalus* in various HCAs, and observed relative inhibition similar to that described here.

Each strain’s ability to metabolize various HCAs was assessed. As in the other assays, there was wide variation between strains, with one consistent feature: all of the strains metabolized caffeic least effectively. No other characteristic was consistent among all strains. Five of the 12 strains metabolized ferulic acid and *p*-coumaric acid to the same degree, and these two HCAs were metabolized between two-fold and three-fold more efficiently than caffeic acid. Of these five strains, three are *B. bruxellensis* and two are *B. anomalus*. All three *B. bruxellensis* strains were previously determined to produce organoleptic qualities inappropriate for brewing, and one of the *B. anomalus* strains fell into this category as well. The other *B. anomalus* strain has been used successfully in a brewery setting. Only strain seven metabolized *p*-coumaric acid more efficiently that ferulic acid. This is a *B. anomalus* strain suitable for brewing.

The remaining six strains all metabolized ferulic acid more efficiently than *p*-coumaric acid. The differences in use of these two HCAs varied widely among these strains, as did the overall level of metabolism. Within this “ferulic acid preference” group are members of both species, and all are able to generate organoleptic qualities suitable for brewing. This correlation is the most striking of the properties investigated for these 12 strains, and is highlighted in Table 3.

Many variables will determine the phenolic compound contribution to the organoleptic qualities of any particular finished beer or wine. The overall concentration and ratio of various HCAs is known to vary widely among different plant species (cereal grains and grapes included), and even between cultivars [12,13]. The proportion of each released into must or wort will vary based on the treatment of the raw materials before and during brewing and winemaking. These properties of raw materials are not generally a consideration when planning a fermentation, and in most cases the HCA values of particular grape harvests and maltings are not known. One exception is the inclusion of a ferulic acid rest to the mash protocol for certain beer styles when clove-like phenols are desired in the final product [29,30]. This 35 °C rest activates feruloyl esterase, releasing ferulic acid from plant cell wall structures into the wort, where Pof+ yeast strains will produce 4-vinylguiacol during fermentation.

Here we investigated the potential of different species and strains of *Brettanomyces* yeast for variable contribution of phenolic compounds during fermentation. Recently, the full genome sequences of one brewing and three wine spoilage strains of *Brettanomyces* have become available [31,32,33,34]. A strain-by-strain sequence alignment of the PAD enzymes reveals a very high degree of amino acid sequence identity (Lentz, unpublished) [35]. Strains varied by zero, two or three amino acids out of 176 total. Interestingly, the highest similarity (100% match across all 176 amino acids) was between one of the spoilage strains and the available Belgian brewery strain. Clearly, more enzyme sequences will be useful in determining the extent to which these small sequence differences contribute to variations in enzyme activity.

It is assumed that the undissociated form of the HCAs can diffuse at an appreciable rate through the plasma membrane into the cell where it is metabolized by the PAD enzyme [36]. Acetic acid can enter *Saccharomyces cerevisiae* yeast cells by this mechanism, but may also enter via large aquaporin channels [37]. There may be an uncharacterized channel or transporter that could add another layer of strain variability influencing phenolic acid metabolism in *Brettanomyces* yeast [38].

Strain variation is only one of several features that contribute to the phenolic character of beer or wine. There is wide variation in the absolute and relative quantities of HCAs in different grape musts and beer worts. This feature may contribute significantly to the observed differences in the contribution of *Brettanomyces* to beer *versus* wine. By controlling for this variable, our study helps to define the specific contribution of PAD enzyme activity to phenolic characteristics of beer and wine.

## 5. Conclusions

We tested brewing and spoilage isolates of Brettanomyces yeast for growth inhibition and metabolism of hydroxycinnamic acids, constituents of both wine must and beer wort. There was wide variability for several of the properties tested, with little correlation to spoilage. We found that strains that metabolized ferulic acid more successfully that p-coumaric acid were all suitable for use in brewing. These results help to define one feature of Brettanomyces’ contribution to the organoleptic qualities of beer and wine.
